# HBx combined with AFB1 triggers hepatic steatosis via COX‐2‐mediated necrosome formation and mitochondrial dynamics disorder

**DOI:** 10.1111/jcmm.14388

**Published:** 2019-07-07

**Authors:** Yuan‐Yuan Chen, Yi Lin, Pei‐Yu Han, Shan Jiang, Lin Che, Cheng‐Yong He, Yu‐Chun Lin, Zhong‐Ning Lin

**Affiliations:** ^1^ State Key Laboratory of Molecular Vaccinology and Molecular Diagnostics, School of Public Health Xiamen University Xiamen China; ^2^ Wuxi School of Medicine Jiangnan University Wuxi China

**Keywords:** aflatoxin B1, cyclooxygenase‐2, hepatic steatosis, hepatitis B virus X protein, mitochondrial dynamics, necrosome

## Abstract

Hepatitis B virus (HBV) infection and aflatoxin B1 (AFB1) exposure have been recognized as independent risk factors for the occurrence and exacerbation of hepatic steatosis but their combined impacts and the potential mechanisms remain to be further elucidated. Here, we showed that exposure to AFB1 impaired mitochondrial dynamics and increased intracellular lipid droplets (LDs) in the liver of HBV‐transgenic mice in vivo and the hepatitis B virus X protein (HBx)‐expressing human hepatocytes both ex vivo and in vitro. HBx combined with AFB1 exposure also up‐regulated receptor interaction protein 1 (RIP1), receptor interaction protein 3 (RIP3) and activated mixed lineage kinase domain like protein (MLKL), providing evidence of necrosome formation in the hepatocytes. The shift of the mitochondrial dynamics towards imbalance of fission and fusion was rescued when MLKL was inhibited in the HBx and AFB1 co‐treated hepatocytes. Most importantly, based on siRNA or CRISPR/Cas9 system, we found that the combination of HBx and AFB1 exposure increased cyclooxygenase‐2 (COX‐2) to mediate up‐regulation of RIP3 and dynamin‐related protein 1 (Drp1), which in turn promoted location of RIP3‐MLKL necrosome on mitochondria, subsequently exacerbated steatosis in hepatocytes. Taken together, these findings advance the understanding of mechanism associated with HBx and AFB1‐induced hepatic necrosome formation, mitochondrial dysfunction and steatosis and make COX‐2 a good candidate for treatment.

## 1 INTRODUCTION

Hepatic steatosis, characterized by aberrant accumulation of lipid droplets (LDs), is suggested to be a prerequisite and cofactor for fatty liver disease and subsequent hepatic injury, such as inflammation, fibrosis, cirrhosis and hepatocellular carcinoma (HCC). In addition to the genetic and diet‐related factors, multiple epidemic studies suggested that hepatic steatosis was common in patient with chronic hepatitis B virus (HBV) infection.^1^ Hepatitis B virus X protein (HBx) is a multifunctional protein encoded by HBV genome. It was reported that HBx trigged lipid accumulation in hepatocytes by increasing de novo lipogenesis and fatty acid (FA) uptake.^2^, ^3^ However, there was a cross‐sectional population study in Hong Kong demonstrated that HBV infection was associated with a lower prevalence of non‐alcoholic fatty liver disease (NAFLD) and hypertriglyceridemia.^4^ This observation agreed with a prior data from Taiwan indicating that HBV‐infected patients had reduced serum triglyceride (TG).^5^ Whether HBV infection correlated with the development of hepatic steatosis is still controversial, and the role of HBx in it remains to be elucidated. Apart from HBV, the liver is a target organ of aflatoxin B1 (AFB1), one of mycotoxin found in the human foods. Exposure to AFB1 had been shown to affect some pathogenic events implicated in hepatic lipid homoeostasis. For example, exposure to 0.5 and 1 mg/kg of AFB1 for 7 days induced liver damage and dysregulation of genes associated with lipid metabolism in rats.^6^ Notably, HBV‐infected patients would be more susceptible to liver injury induced by AFB1.^7^ However, just one study in zebrafish showed that combination of HBx and AFB1 promoted hepatic steatosis and increased the expression of lipogenic factors and genes related to lipid metabolism yet.^8^ It is necessary to further determine the combined impact of HBx and AFB1 exposure on hepatic lipid homoeostasis and the potential mechanisms.

Increasing number of studies suggested that alterations of mitochondrial energetic metabolism were the initiating event in the development and progression of hepatic steatosis.^9^ Thus, fatty liver disease has been considered as a mitochondrial related disease.^10^ Moreover, mitochondria are considered to be one of cell death executor and several pathways of programmed cell death are involved in regulating liver homoeostasis.^11^ Necroptosis, a mode of programmed cell death, is involved in the development of NAFLD.^12^ Although aberrant mitochondrial dynamics and function were served as crucial pathophysiological factors in HBV‐infected hepatocytes^13^ and AFB1 could also impair mitochondrial functions,^14^ study addressing the interactive relations of mitochondrial dysfunction and necroptosis in AFB1 exposure and/or HBV infection‐driven hepatic steatosis is lacking up to now.

Interestingly, our previous study showed that cyclooxygenase‐2 (COX‐2) promoted mitochondrial translocation of dynamin‐related protein 1 (Drp1), an important mediator of mitochondrial fission, resulting in a higher percentage of fragmented mitochondria in the cancer stem cells.^15^ In spite of the extremely low expression in the liver, COX‐2 can be induced by a variety of extracellular and intracellular stimuli, including reactive oxygen species, chemicals and viral infections.^16^ Higher expression of COX‐2 was observed in patients with clinical diagnosis of NAFLD than those in subjects with histologically normal liver.^17^ More importantly, knocking down of COX‐2 with small interfering RNA for prostaglandin‐endoperoxide synthase 2 gene (*PTGS2*) encoding COX‐2 (si*PTGS2*) or selective COX‐2 inhibitor celecoxib ameliorated steatosis in palmitate‐treated hepatocytes and in liver of high‐fat diet‐fed rats,^18^ highlighting that COX‐2 should be a key regulator in the development of NAFLD. As we known, higher expression of COX‐2 was shown in the hepatocytes of HBV‐infected patients, even in patients with antiviral therapies.^19^ HBx decreased mitochondrial membrane potential and ATP, and up‐regulated COX‐2 in vitro.^20^ Moreover, the expression of COX‐2 was also increased in the liver of AFB1‐treated rats and chicken.^21^, ^22^ Therefore, there seemed to be a close, yet not totally understood, and complex interaction among COX‐2, mitochondrial dysfunction, necrosome formation and lipid accumulation in hepatocytes co‐exposed to HBx and AFB1. And the aim of this study was to figure out whether HBx and AFB1 could act in concert to activate COX‐2, subsequently alter mitochondrial dynamics and facilitate necrosome formation, which ultimately trigger hepatic steatosis.

## 2 MATERIALS AND METHODS

### 2.1 Cell culture and treatment

Three HBx‐expressing cell lines including HepG2.2.15 cells integrating two head‐to‐tail copies of the HBV genome, HBx expressing HepG2 cells (HepG2‐Tet‐ON‐HBx) and differentiated HepaRG cells were selected and constructed (Supporting information: Material and methods).^23^ All these stable cell lines expressing HBx were treated with 1 μmol/L of AFB1 (Sigma‐Aldrich, MO) for 48 or 72 hours. AFB1 was dissolved in DMSO. For COX‐2 and Drp1 knockdown, the cells were transfected with 50 nmol/L annealed double‐stranded si*PTGS2* and siRNA for dynamin 1 like gene (*DNM1L*) encoding Drp1 (si*DNM1L*) (Ribobio, Guangzhou, China) using Lipofectamine® 2000 (Invitrogen, CA), respectively. COX‐2 knockdown cell line was also generated by CRISPR/Cas9 systems (Supporting information: Material and methods).

### 2.2 Animals and treatment

All of the animal experiments were performed in accordance with the guidelines of the Xiamen University Institutional Committee for the Care and Use of Laboratory Animals (no. XMULAC20140033). Mice were housed under specific pathogen‐free conditions and had ad libitum access to food and water. The human‐liver‐chimeric (HLC) mice for HBV infection were constructed through transplantation of primary human hepatocytes (PHHs) into Fah^−/−^/Rag2^−/−^/Il2rg^−/−^ (FRG) male mice according to Azuma's methods.^24^ FRG male mice were infected with HBV derived from the supernatant of HepaAD38 cells for 6 weeks. Then, PHHs were isolated from the mice and exposed to 1 μmol/L of AFB1 for 48 or 72 hours. The HBV‐transgenic (HBV‐Tg) mice were produced via the microinjection of pAAV/HBV1.2 (genotype Ae) into the embryos of male C57BL/6 mice. Male HBV‐Tg and C57BL/6 mice were administered peanut oil or 5 mg/kg of AFB1 dissolved in peanut oil by intraperitoneal injection on the first, fourth and seventh day for a total three injections.

### 2.3 Gene expression omnibus (GEO) data analysis

Gene expression data of HBV‐infected patients were searched from GEO database (http://www.ncbi.nlm.nih.gov/geo/). The relative gene expressions of *DNM1L*, *PTGS2* and *MLKL* were analysed as previously described.^15^ Correlations between the expression of *DNM1L* and *PTGS2*, as well as *PTGS2* and *MLKL*, were evaluated by Pearson's correlation analysis (SPSS, IL).

### 2.4 Immunofluorescence (IF) and immunohistochemistry (IHC) analysis

For mitochondria morphology, the cells were incubated with 100 nmol/L MitoTracker Red (Thermo Scientific, MA) for 30 min at 37°C. Fluorescence images of cells were obtained with a laser‐scanning confocal microscope (Zeiss LSM 780, Jena, Germany). Mitochondrial morphological characteristics were quantified by the mitochondrial fragmentation counts as described previously.^15^ For IF, the cells were fixed in 4% paraformaldehyde and permeabilized with 0.5% Triton X‐100. Then the cells were blocked with 1% bovine serum albumin (BSA) and incubated with corresponding primary antibodies. Finally, the cells were incubated with secondary antibody and were imaged using laser‐scanning confocal microscope (Leika SP8, Wetzlar, Germany). Quantification of co‐localization was analysed by Image Pro Plus (IPP) version 6.0 software (MD, USA). For IHC, liver sections were stained with antibodies against HBx, COX‐2, RIP3 and p‐Drp1^Ser616^. All IHC images were examined under an upright light microscope (Nikon, Tokyo, Japan). The information of antibodies was described in the Supporting information: Material and methods.

### 2.5 Proximity ligation assay (PLA)

The protein interaction between COX‐2 and RIP3 was determined by PLA using a Duolink® In Situ Detection Reagents (Sigma‐Aldrich) as previously described (Supporting information: Material and methods).^15^

### 2.6 Co‐immunoprecipitation (Co‐IP)

HepG2‐Tet‐ON‐HBx cells were lysed with Nonidet P 40 (NP40) lysis buffer (Sigma‐Aldrich) and the whole cell extracts were collected. To precipitate the target protein, the cell lysates were incubated with SureBead (BioRad, CA) previously coated with anti‐COX‐2 antibody at room temperature for 1 hour. After the washing steps with PBS supplemented with 0.1% Tween 20, the binding proteins on the beads were eluted with 1× Sodium dodecyl dulfonate (SDS) buffer by 5‐min boiling. Eluted proteins were analysed by Western blot.

### 2.7 Lipid index assays

The liver sections of mice were stained by Oil‐Red‐O (Jiancheng, Nanjing, China) to visualize LDs and the quantification was performed using IPP 6.0 software. The lipid content in cultured cells were also visualized and quantified using Oil‐Red‐O staining. In addition, lipids in the liver of mice were extracted and the hepatic TG and total cholesterol (TC) were measured using commercial kits (Jiancheng) according to the manufacturer's instructions.

### 2.8 Western blot analysis

The livers and the cells were harvested and lysed using radio immunoprecipitation assay (RIPA) buffer (Beyotime, Jiangsu, China) or 1× SDS‐PAGE loading buffer. A mitochondrial isolation kit (EnzoLife, PA) were used to separate mitochondrial and cytosolic fractions. The protein concentrations were measured using BCA protein assay kit (Beyotime). For western blots, equal amounts of protein were subjected to gel electrophoresis, transferred to polyvinylidene fluoride (PVDF) membranes, and blocked with 5% skimmed milk before being incubated with primary antibodies (Supporting information: Material and methods). Then, the membranes were incubated with secondary antibodies and were visualized using the ECL‐Plus chemiluminescence reagent (Advansta, CA, USA).

### 2.9 RNA extraction and quantity real‐time PCR (qRT‐PCR)

Total RNA was extracted using TRIzol reagent (Takara, Osaka, Japan) and was reverse transcribed with Prime ScriptTM RT reagent kit (Takara). qRT‐PCR was then performed with SYBR® Premix Ex Taq™ II Kit (Takara) by CFX96 Touch^TM^ Detection System (Bio‐Rad, CA). The primer sequences were listed in Supporting information: Table S1.

### 2.10 Statistics

All data were expressed as the mean ± standard deviation (SD). All statistical analyses were conducted with SPSS software v.16.0. The differences in data measurements among the groups were analysed initially by a homogeneity test of variances, and then, either an unpaired Student's *t *test or one‐way ANOVA was performed under equivalent conditions. Pearson's correlation analysis was conducted for variables correlation. *P* < 0.05 was considered to be statistically significant.

## 3 RESULTS

### 3.1 Exposure to AFB1 exacerbated lipid droplets (LDs) accumulation in the liver of HBV‐Tg mice and HBx‐expressing hepatocytes

**Figure 1:**
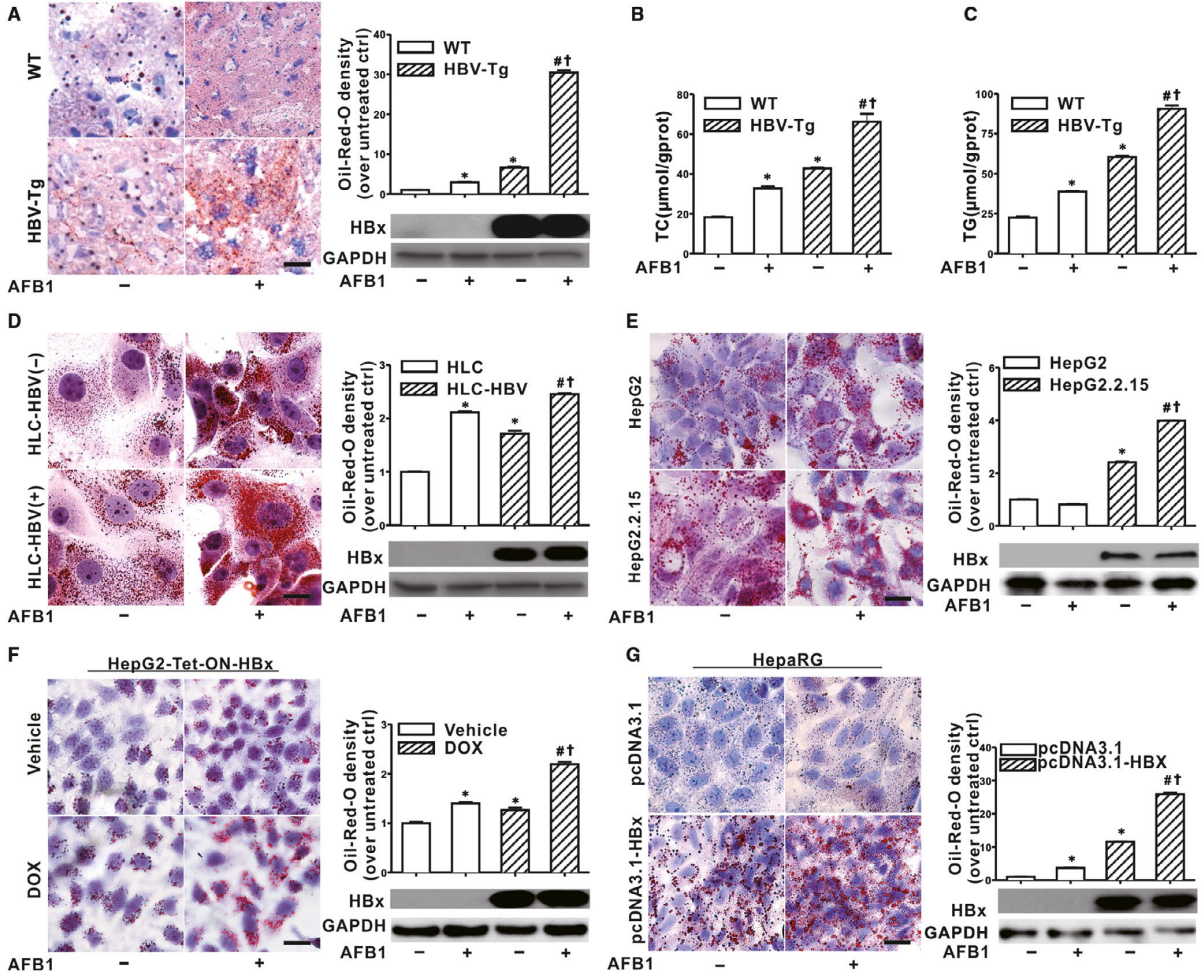
Exposure to aflatoxin B1 (AFB1) exacerbated lipid droplet (LD) accumulation in the liver of HBV‐Tg mice and hepatitis B virus X protein (HBx)‐expressing hepatocytes. A‐C, HBV‐Tg or WT mice were treated with AFB1 or vehicle (peanut oil) every 3 days for 1 week. Representative images and spectrophotometric quantification of LDs in liver section stained with Oil‐Red‐O (A). Levels of hepatic TC (B) and TG (C). D, Representative Oil‐Red‐O staining images and spectrophotometric quantification of LDs in PHHs ex vivo. PHHs were isolated from the HLC mice with (HLC‐HBV(+)) or without (HLC‐HBV(‐)) HBV infection and exposed to 1 μmol/L of AFB1 or DMSO for 72 h. E‐G, 1 μg/mL of DOX were used to stimulate HBx expression. Differentiated HepaRG cells were transfected with pcDNA3.1‐HBX (1 μg/mL) to induce HBx expression. Cells were exposed to 1 μmol/L of AFB1 or DMSO for 72 h. Representative images and spectrophotometric quantification of LDs in HepG2.2.15 cells (E), HepG2‐Tet‐ON‐HBx cells (F) and differentiated HepaRG cells (G). Scale bar represents 10 μm. Data were represented by mean ± SD. n = 3. *P < 0.05 compared with unexposed cells or WT mice. ^#^ *P* < 0.05 compared with HBx‐expressing cells or HBV‐Tg mice. ^†^ *P* < 0.05 compared with AFB1‐exposed cells or AFB1‐exposed WT mice

Based on the Oil‐Red‐O staining, Figure 1A showed that the amount of LDs was modestly increased in the liver tissues of HBV‐Tg mice compared with those in wildtype (WT) mice; and significantly higher levels of LDs were observed in the liver of HBV‐Tg mice exposed to AFB1 than those in the unexposed HBV‐Tg mice. Meanwhile, hepatic TG and TC contents were increased in the AFB1‐exposed WT mice, the HBV‐Tg mice, as well as the AFB1‐exposed HBV‐Tg mice compared with corresponding controls (Figure 1B, C). Besides animal models, human hepatocytes are the natural target of HBV infection. HLC mice for HBV infection were constructed and the PHHs were exposed to AFB1 (Supporting information: Figure S1). Figure 1D showed that LDs were increased in AFB1‐exposed PHHs from HLC mice compared with unexposed PHHs, no matter whether infected HBV. The highest amounts of LDs were observed in the AFB1‐exposed‐PHHs from HBV‐infected HLC mice (Figure 1D). Consistent with the in vivo and ex vivo results, LDs were increased in the HepG2.2.15 cells constitutively replicating HBV, no matter whether exposed to AFB1 (Figure 1E). After exposed to AFB1, LDs were significantly increased in both the HepG2‐Tet‐ON‐HBx cells (Figure 1F) and the differentiated HepaRG cells (Figure 1G). Taken together, HBx combined with AFB1 exposure triggered LDs accumulation in the hepatocytes.

### 3.2 Drp1‐dependent mitochondrial dynamics abnormalities regulated HBx and AFB1‐induced hepatic steatosis

**Figure 2:**
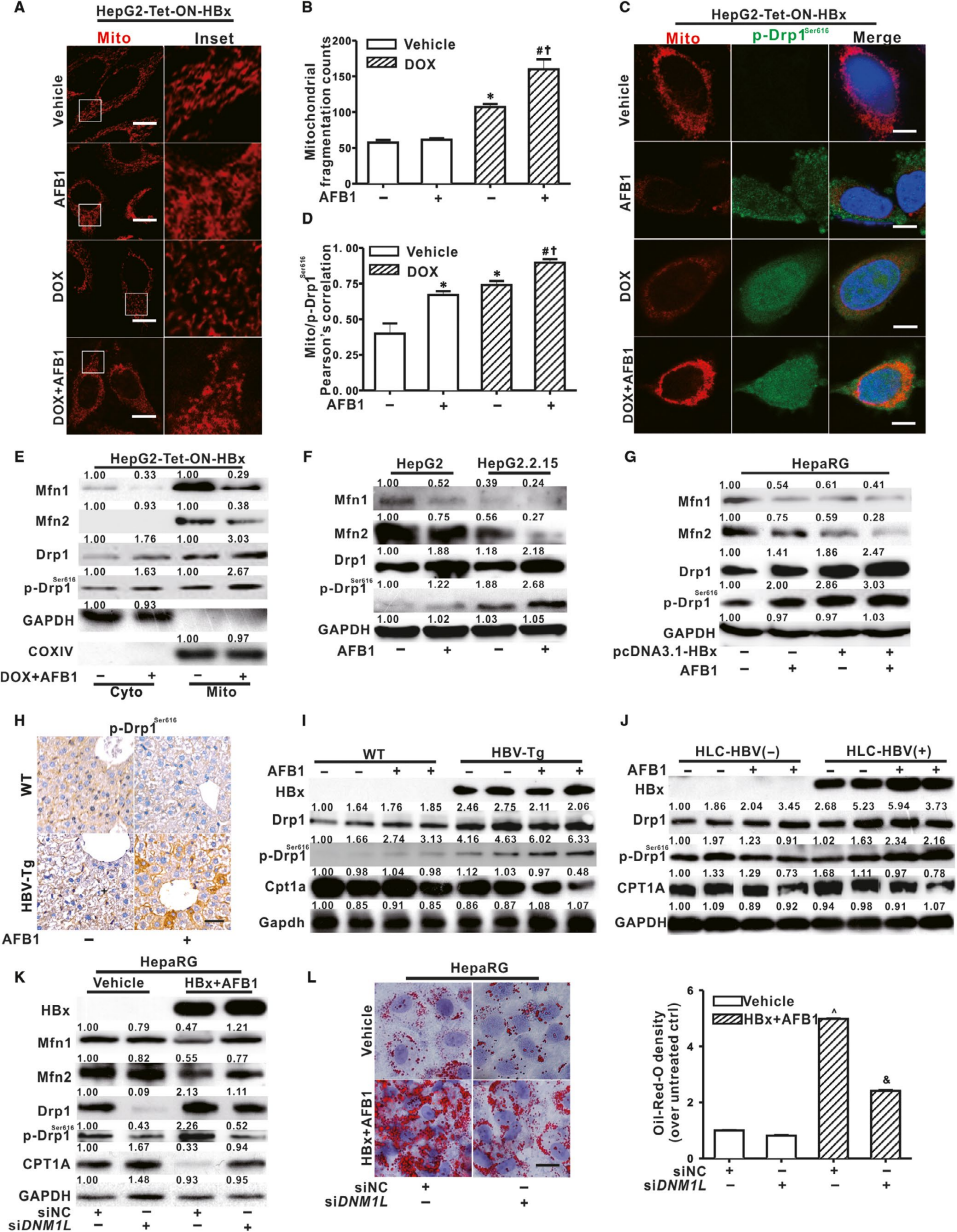
Drp1‐dependent mitochondrial dynamics abnormalities regulated hepatitis B virus X protein (HBx) and aflatoxin B1 (AFB1)‐induced hepatic steatosis. A, Representative immunofluorescence images of mitochondria in HepG2‐Tet‐ON‐HBx cells stained with MitoTracker. Scale bar represents 10 μm. B, Quantification of mitochondrial fragmentation counts analysed by Image Pro Plus version 6.0 software. C, Representative images of the immunofluorescence co‐staining for p‐Drp1^Ser616^ (Green) and MitoTracker (Red) in HepG2‐Tet‐ON‐HBx cells. Nucleus was stained with DAPI (blue). Scale bar represents 10 μm. D, Pearson’s correlation for the co‐localization of p‐Drp1^Ser616 ^with mitochondria. E, The expression of mitochondrial dynamics‐related proteins in mitochondrial and cytosolic fractions of HepG2‐Tet‐ON‐HBx cells. GAPDH and COXIV were served as the loading control of cytosolic and mitochondrial fraction, respectively. F and G, The protein expression of mitochondrial dynamics‐related proteins in whole cell lysates of HepG2.2.15 cells (F), and HepaRG cells (G). H, Immunohistochemical staining of p‐Drp1^Ser616^ in the liver of WT and HBV‐Tg mice treated with AFB1 or vehicle. Scale bar represents 50 μm. I, The protein expression of p‐Drp1^Ser616^, Drp1 and Cpt1a in the liver of WT and HBV‐Tg mice. J, The protein expression of p‐Drp1^Ser616^, Drp1 and CPT1A in PHHs from livers of HLC mice. K and L, The differentiated HepaRG cells were transfected with siNC or si*DNM1L* (50 nmol/L) for 12 h before AFB1 treatment and HBx expression. K, The protein expression of mitochondrial dynamics‐related proteins and CPT1A in differentiated HepaRG cells. L, Representative images and spectrophotometric quantification of lipid droplets (LDs) in differentiated HepaRG cells. Scale bar represents 10 μm. For western blot analysis, GAPDH served as a loading control. Data were represented by mean ± SD. n = 3. *P < 0.05 compared with unexposed HepG2‐Tet‐ON‐HBx cells. ^#^ *P* < 0.05 compared with DOX‐treated HepG2‐Tet‐ON‐HBx cells. ^†^ *P* < 0.05 compared with AFB1‐exposed HepG2‐Tet‐ON‐HBx cells. ^^^ *P* < 0.05 compared with unexposed differentiated HepaRG cells transfected with siNC. ^&^ *P* < 0.05 compared with HBx and AFB1‐cotreated differentiated HepaRG cells transfected with siNC

Given that mitochondrial dysfunction is a key mediator of hepatic lipid metabolism, the impact of HBx and AFB1 on mitochondrial morphology and dynamics was investigated. MitoTracker staining showed that the mitochondrial network was broken down and fragmented into short rods or spheres in HepG2‐Tet‐ON‐HBx cells when HBx were switched on by DOX (Figure 2A,B). Mitochondrial fragmentation was further aggravated after AFB1 exposure in these HBx‐expressing HepG2‐Tet‐ON‐HBx cells (Figure 2A,B). Mitochondrial fragmentation could be results of activated fission, suppressed fusion or both.^25^ Figure 2C,D showed that combined exposure to HBx and AFB1 induced an accumulation of p‐Drp1^Ser616^, one of the main proteins implicated in mitochondrial fission, in the mitochondria. This result was consistent with the western blot analysis that the protein expression of p‐Drp1^Ser616^ was increased in the mitochondrial fractions in DOX‐induced and AFB1‐treated HepG2‐Tet‐ON‐HBx cells (Figure 2E). Moreover, increased p‐Drp1^Ser616^ was also observed in other HBx and AFB1 co‐exposed hepatic cell lines, including HepG2.2.15 and differentiated HepaRG cells (Figure 2F,G). In contrast to p‐Drp1^Ser616^, decreased expression of mitochondrial fusion protein mitofusin 1 and 2 (Mfn1 and Mfn2) was observed in HBx‐expressing HepG2‐Tet‐ON‐HBx, HepG2.2.15, and differentiated HepaRG cells compared with corresponding controls (Figure 2E‐G). Moreover, AFB1 aggravated down‐regulation of Mfn1 and Mfn2 induced by HBx in these cells (Figure 2E‐G). In line with the in vitro experiments, exposure to AFB1 increased expression of p‐Drp1^Ser616^ in both the liver of HBV‐Tg mice in vivo (Figure 2H,I) and PHHs from the livers of HLC mice with HBV infection ex vivo (Figure 2J), compared with corresponding controls. Decreased protein expression of carnitine palmitoyltransferase 1A (CPT1A) (Figure 2I,J), a primary rate‐limiting enzyme involved in mitochondrial FA β‐oxidation, occurred in parallel to the changes in mitochondrial dynamics. To further demonstrate that inhibition of CPT1A observed in HBx and AFB1‐exposed hepatocytes were mediated through altering Drp1‐dependent mitochondrial dynamics, Drp1 was knocked down by si*DNM1L* in differentiated HepaRG cells. Figure 2K,L showed that HBx and AFB1 co‐treatment failed to decrease CPT1A, Mfn1 and Mfn2, and to increase LDs when Drp1 was knocked down in differentiated HepaRG cells. As a result, Drp1‐associated mitochondrial dynamics abnormalities were involved in the suppressing of CPT1A during HBx and AFB1 co‐treatment‐induced LDs accumulation.

### 3.3 COX‐2 regulated hepatic steatosis via mitochondrial dynamics remodeling in AFB1‐treated HBV‐Tg mice and HBx‐expressing hepatocytes

**Figure 3:**
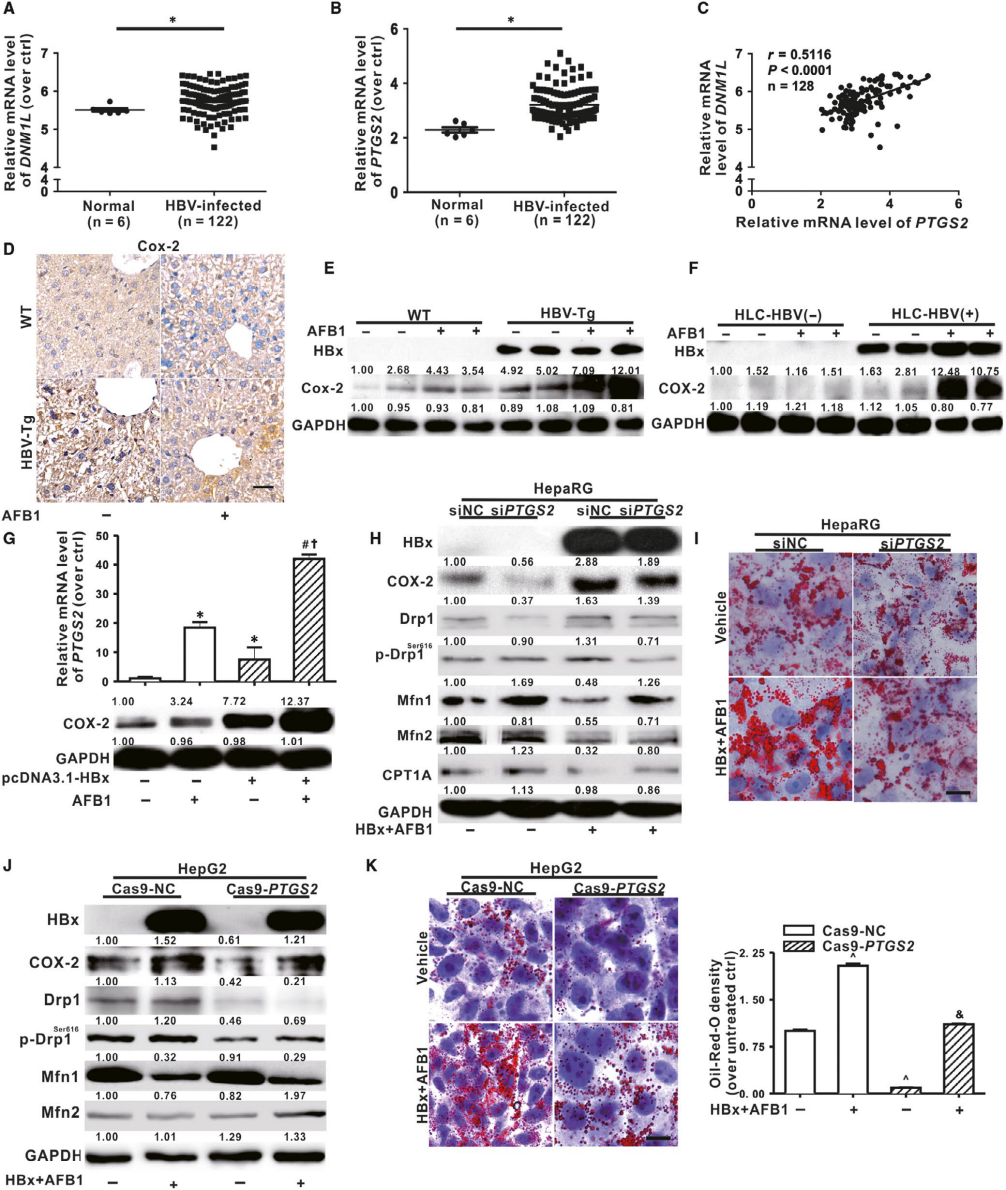
COX‐2 regulated hepatitis B virus X protein (HBx) and aflatoxin B1 (AFB1)‐induced hepatic steatosis. A and B, In a cohort from GEO database (accession no. GSE83148), the mRNA level of *DNM1L* (A) and *PTGS2* (B) was analysed in the liver tissues from CHB patients (n = 122) and normal participants (n = 6). C, Pearson’s correlation analysis between the mRNA expression of *DNM1L* and *PTGS2*. D, Immunohistochemical staining of Cox‐2 in the liver of WT and HBV‐Tg mice treated with AFB1 or vehicle. Scale bar represents 50 μm. E, The protein levels of Cox‐2 in the liver of WT and HBV‐Tg mice. F, The protein levels of COX‐2 in the liver of PHHs from livers of HLC mice. G, The mRNA and protein levels of COX‐2 in differentiated HepaRG cells. H and I, Differentiated HepaRG cells were transfected with siNC or si*PTGS2* (50 nmol/L) for 12 h before AFB1 treatment and HBx expression. H, The expression of mitochondrial dynamics‐related proteins and CPT1A in differentiated HepaRG cells. I, Representative images and spectrophotometric quantification of lipid droplets (LDs) in differentiated HepaRG cells. Scale bar represents 10 μm. J and K, COX‐2‐deficient HepG2 cells were generated via CRISPR/Cas9 system. The HepG2‐Cas9‐NC and HepG2‐Cas9‐*PTGS2* cells were transfected with pcDNA3.1‐HBX (1 μg/mL) to induce HBx expression and the cells were exposed to 1 μmol/L of AFB1 or DMSO for 48 h. J, The expression of mitochondrial dynamics‐related proteins. K, Representative images and spectrophotometric quantification of LDs. Scale bar represents 10 μm. For Western blot, GAPDH was served as a loading control, and for mRNA expression, *ACTB* was served as a loading control. Data were represented by mean ± SD. n = 3. *P < 0.05 compared with unexposed differentiated HepaRG cells. ^#^ *P* < 0.05 compared with HBx‐expressing differentiated HepaRG cells. ^†^ *P* < 0.05 compared with AFB1‐exposed differentiated HepaRG cells. ^^^ *P* < 0.05 compared with HepG2‐Cas9‐NC cells. ^&^ *P* < 0.05 compared with HBx and AFB1‐cotreated HepG2‐Cas9‐NC cells

The role of COX‐2 in HBV‐related mitochondrial dynamics abnormalities was investigated. Genomic expression data set of HBV infected patient were employed to explore the relationship among expression of *DNM1L*, *PTGS2* and HBV infection. Figure 3A,B showed that higher mRNA levels of *DNM1L* and *PTGS2* was shown in the liver biopsy of HBV‐infected patient tissues (n = 122) compared with those in normal tissues (n = 6). Moreover, there was a significant correlation (r = 0.5116, *P* < 0.0001) between expression of *DNM1L* and *PTGS2* in these samples (Figure 3C). Figure 3D,E showed that protein levels of Cox‐2 was increased in the liver of both AFB1‐exposed mice and HBV‐Tg mice compared with corresponding control mice, and the highest level was shown in the liver of AFB1‐exposed HBV‐Tg mice in vivo. In addition, significantly elevated COX‐2 was observed in AFB1‐exposed PHHs from the livers of HLC mice with HBV infection ex vivo (Figure 3F) and human hepatic cell lines following single and combined treatment of HBx and AFB1 in vitro (Figure 3G, Supporting information: Figure S2). Importantly, HBx and AFB1‐stimulated up‐regulation of Drp1 and down‐regulation of CPT1A, Mfn1 and Mfn2 were ameliorated when differentiated HepaRG cells were transfected with si*PTGS2* (Figure 3H), consequently increasing LDs deposits (Figure 3I). Besides siRNA, CRISPR/Cas9 gene‐editing technique was applied to generate COX‐2‐deficient HepG2‐Cas9‐*PTGS2* cell line. Figure 3J,K confirmed that these inducible effects of HBx and AFB1 on mitochondrial dynamics imbalance and subsequently LDs accumulation were indeed abolished in HepG2‐Cas9‐*PTGS2* cells. Taken together, COX‐2 was critical in mediating Drp1‐dependent mitochondrial fission during HBx and AFB1‐induced hepatic steatosis.

### 3.4 HBx and AFB1‐induced steatosis was associated with the formation of necrosome

**Figure 4:**
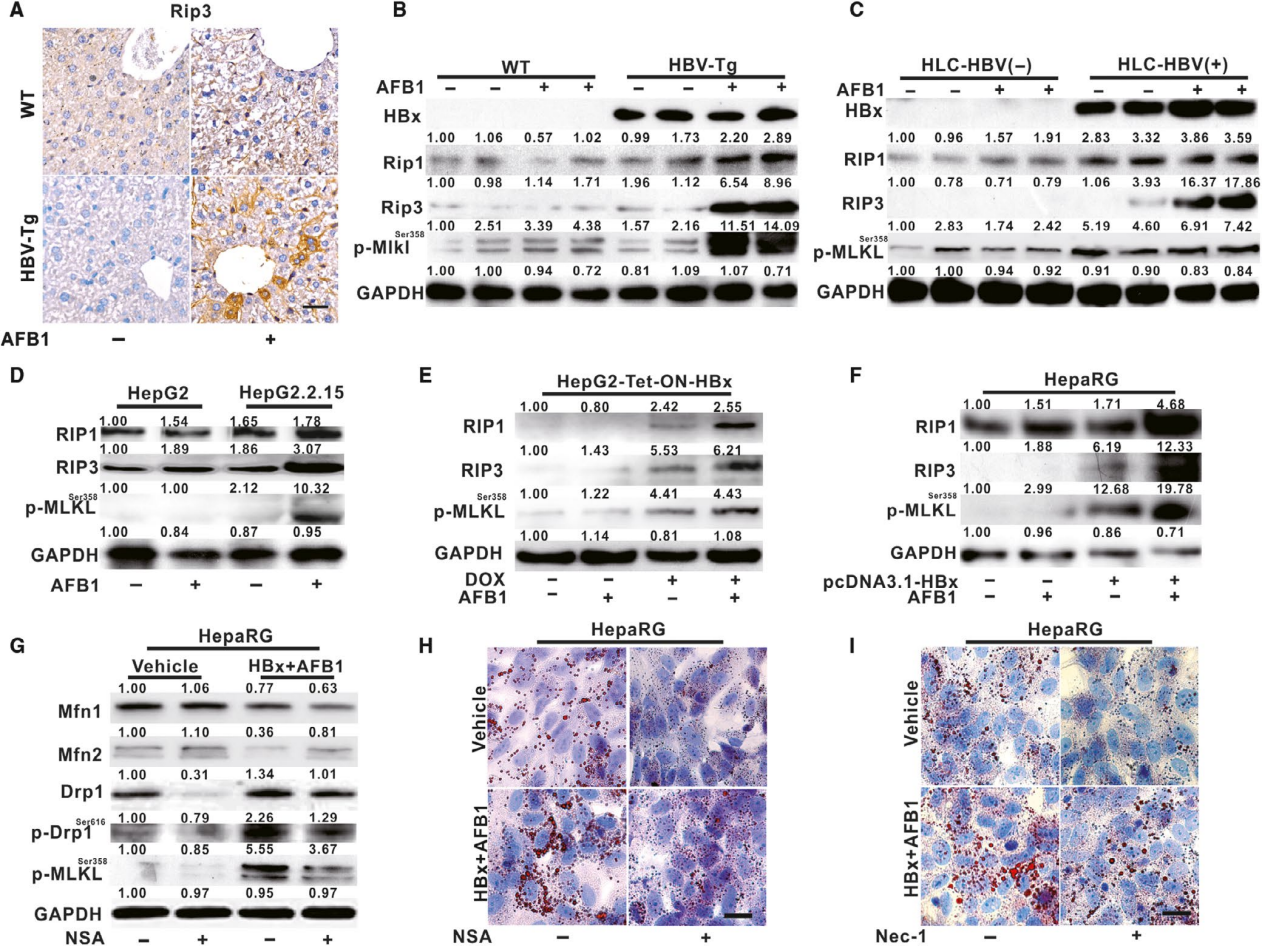
Hepatitis B virus X protein (HBx) and aflatoxin B1 (AFB1)‐induced hepatic steatosis was associated with necrosome formation. A, Immunohistochemical staining of Rip3 in the liver of WT and HBV‐Tg mice treated with AFB1 or vehicle. Scale bar represents 50 μm. B, The protein expression of the necrosome components in the liver of WT and HBV‐Tg mice. C, The protein levels of the necrosome components in PHHs from livers of HLC mice. D‐F, The protein levels of the necrosome components in HepG2.2.15 cells (D), HepG2‐Tet‐ON‐HBx cells (E), and differentiated HepaRG cells (F). G‐I, Differentiated HepaRG cells were pretreated with 1 μmol/L NSA (G and H) or 15 μmol/L Nec‐1 (I) for 12 h before HBx and AFB1 treatment. G, The expression of mitochondrial dynamics‐related proteins in differentiated HepaRG cells with NSA treatment. H and I Representative images of lipid droplets (LDs) in differentiated HepaRG cells with NSA (H) and Nec‐1 (I) treatment. Scale bar represents 10 μm. For western blot analysis, GAPDH was served as a loading control

Mitochondrial dynamics imbalance has been reported to be involved in cell apoptosis, necroptosis, or other regulatory cell death. Figure 4A‐C showed that the protein expression of both RIP1 and RIP3, as well as phosphorylation of MLKL (p‐MLKL), a substrate of RIP kinase, were increased in the liver of AFB1‐exposed HBV‐Tg mice and AFB1‐exposed PHHs from mice with HBV infection, compared with unexposed corresponding controls. Consistent with the results shown in vivo and ex vivo, up‐regulation of the RIP3‐MLKL signalling cascades were observed in three AFB1‐exposed hepatocytes with HBx expression (Figure 4D‐F). Interestingly, Figure 4G showed that dysregulation of Drp1, Mfn1 and Mfn2 in response to HBx and AFB1 in differentiated HepaRG cells were reversed by MLKL inhibitor necrosulfonamide (NSA). What's more, pretreatment of NSA (Figure 4H) or necrostatin‐1 (Nec‐1) (Figure 4I), an inhibitor of RIP1, also counteracted the increase in LDs in the HBx and AFB1 co‐exposed differentiated HepaRG cells. HBx and AFB1‐induced necrosome formation was responsible, at least in part, for the hepatic mitochondrial dynamics imbalance and related LDs accumulation.

### 3.5 HBx and AFB1‐induced formation of necrosome depended on the COX‐2

**Figure 5:**
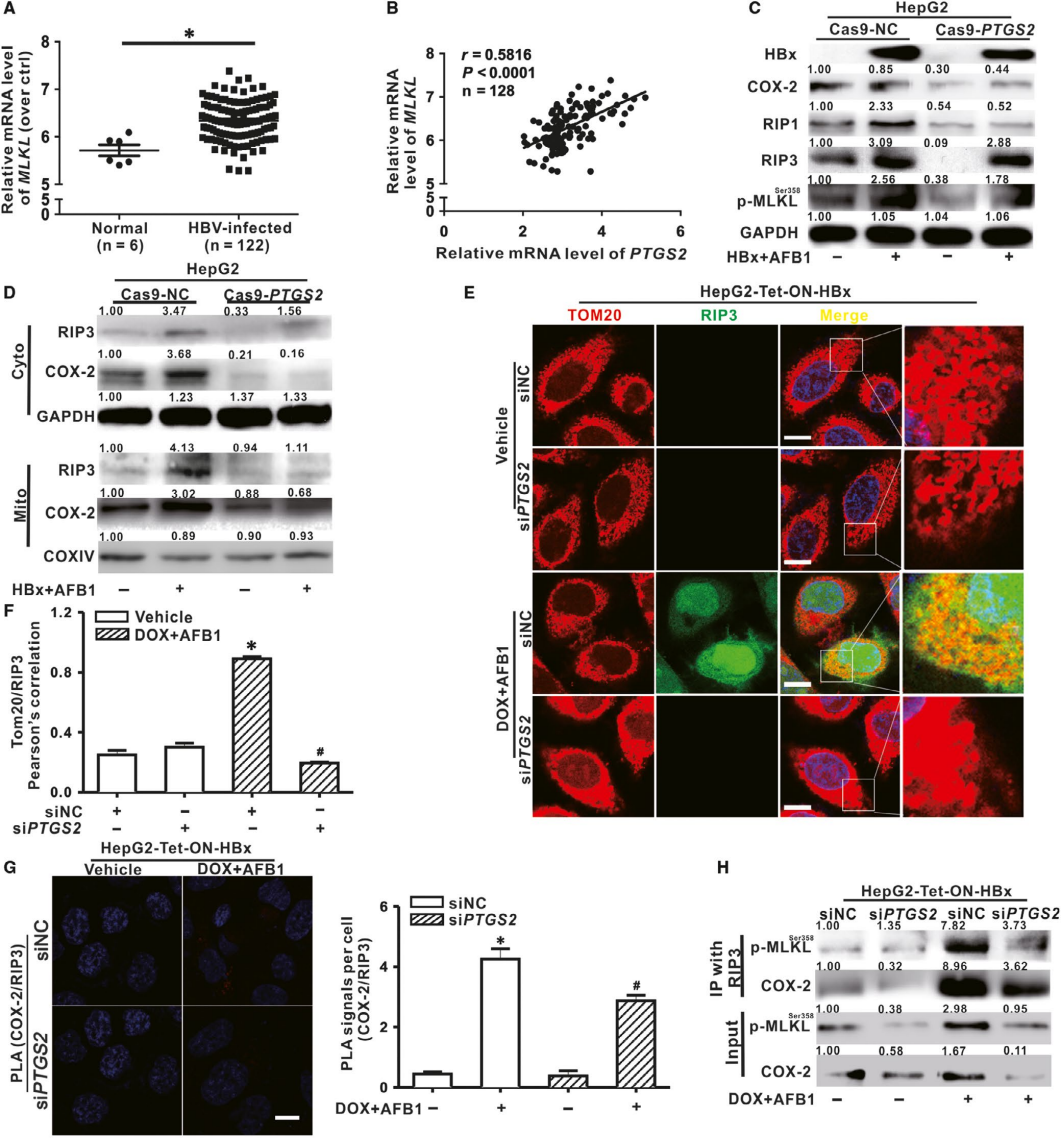
Hepatitis B virus X protein (HBx) and aflatoxin B1 (AFB1)‐induced formation of necrosome depended on the mitochondrial COX‐2 expression. A, In a cohort from GEO database (accession no. GSE83148), the mRNA level of *MLKL* was analysed in the liver tissues from CHB patients (n = 122) and in normal participants (n = 6). B, Pearson’s correlation analysis between the mRNA expression levels of *PTGS2* and *MLKL*. C, The protein expression of COX‐2 and the necrosome components in HepG2‐Cas9‐NC and HepG2‐Cas9‐*PTGS2* cells. D, The protein expression of RIP3 and COX‐2 in mitochondrial and cytosolic fractions of HepG2‐Cas9‐NC and HepG2‐Cas9‐*PTGS2* cells. E‐H, HepG2‐Tet‐ON‐HBx cells were transfected with siNC or si*PTGS2* (50 nmol/L) for 12 h before DOX stimulation and AFB1 treatment. E and F, Representative images (E) and spectrophotometric quantification (F) of the immunofluorescence co‐staining for TOM20 (Red) and RIP3 (Green) in HepG2‐Tet‐ON‐HBx cells. G, The interaction of COX‐2 with RIP3 was detected by proximity ligation assay (PLA). Interaction events were shown as red foci. Quantification of COX‐2/RIP3 interaction events were shown on the right side of the bar. H, Immunoblot analysis of the interaction of RIP3 with COX‐2 or p‐MLKL in immunoprecipitates of HepG2‐Tet‐ON‐HBx cells. GAPDH and COXIV were served as the loading control of cytosolic and mitochondrial fraction, respectively. Scale bar represents 10 μm. Data were represented by mean ± SD. n = 3. *P < 0.05 compared with untreated HepG2‐Tet‐ON‐HBx cells transfected with siNC. ^#^ *P* < 0.05 compared with DOX and AFB1‐cotreated HepG2‐Tet‐ON‐HBx cells transfected with siNC

We showed that HBx and AFB1‐triggerd mitochondrial dynamics abnormalities could be downstream of COX‐2 and necrosome formation on mitochondria, but whether COX‐2 was involved in the formation of necrosome remained unclear. Figure 5A showed that the mRNA expression of *MLKL* was higher in the liver of HBV‐infected patient than those in normal tissues, and moreover, the expression of *MLKL* was positively correlated with *PTGS2* (Figure 5B), suggesting that COX‐2 might have a role in regulation of necrosome formation. As expected, inhibition of COX‐2 expression by the CRISPR/Cas9 system decreased the HBx and AFB1‐stimulated up‐regulation of RIP1, RIP3 and p‐MLKL (Figure 5C). The level of RIP3 in mitochondrial was also decreased when COX‐2 was inhibited in HBx and AFB1 co‐treated HepG2 cells (Figure 5D) and HepG2‐Tet‐ON‐HBx cells (Figure 5E,F). More importantly, PLA showed that combined exposure to HBx and AFB1 significantly increased the interaction between COX‐2 and RIP3 in HepG2‐Tet‐ON‐HBx cells, whereas the interaction was reduced when COX‐2 was inhibited by si*PTGS2* (Figure 5G). Furthermore, co‐treatment of HepG2‐Tet‐ON‐HBx cells with HBx and AFB1 promoted binding of RIP3 to COX‐2 and MLKL, which suggested that HBx and AFB1 triggered the formation of a COX‐2‐RIP3‐MLKL complex on mitochondria. When COX‐2 was inhibited, the interaction between RIP3 and MLKL was weakened (Figure 5H), subsequently blocked the necrosome formation and protected hepatocytes from necroptosis. Therefore, HBx and AFB1 mediated COX‐2 up‐regulation was required for MLKL to be recruited to RIP3 for subsequent execution of necroptosis, and COX‐2 could serve as a potential therapeutic target for necrotic cell death‐associated hepatic steatosis.

## 4 DISCUSSION

HBV is a small hepatotropic DNA virus responsible for some chronic liver diseases in humans, but the role of HBV infection in the fatty liver diseases remains controversial.^3^, ^4^, ^26^ In this study, together with previous animals and cellular data,^2^, ^26^ the ectopic fat storage was consistently observed in the liver of HBV‐Tg mice. Moreover, we further demonstrated that the amount of LDs was increased in multiple HBx‐expressing human hepatic cell lines including HepG2.2.15, HepG2‐Tet‐ON‐HBx and differentiated HepaRG cells. Given that single use of systems based on liver cell lines existed some inevitable limitations, HLC mice were introduced and the consistent results obtained in PHHs from HBV‐infected HLC mice strengthened the evidence linking HBV infection with hepatic steatosis. In addition, this study showed an effect of AFB1 on exacerbation of HBx‐related hepatic steatosis. AFB1 alone has been suggested as a risk factor for NAFLD.^6^, ^27^ Human are exposed to AFB1 mainly through contaminated food, especially corns, peanuts and rice. After ingestion, AFB1 is activated by cytochrome P450 within the liver microsome system to produce large amount of toxic metabolite.^28^, ^29^ HBV infection has been reported to facilitate metabolism and bioactivation of AFB1 in the liver via the transactivation of pregnane X receptor (PXR) and induction in CYP3A4.^30^, ^31^ Moreover, HBV originating from chronic hepatitis or the virus itself could exacerbate host response to AFB1 and down‐regulated detoxification‐related proteins,^32^, ^33^, ^34^ making HBV‐infected hepatocytes more susceptible to AFB1 toxicity. Instead, exposure to AFB1 would also exacerbate and accelerate the disease progression of HBV infection, perhaps via affecting susceptibility to infection or viral replication.^35^ In tropical and humid area such as Southern China, many people are chronically infected with HBV while also exposing AFB1 in their diet.^7^ Individuals with HBV infection were much more susceptible to AFB1‐associated HCC risk compared with those without these aetiological factors.^36^ Consistent with the combined role of HBV infection and AFB1 exposure in hepatocarcinogenesis, this study highlighted the effects of acute co‐exposure to HBx and AFB1 on hepatic steatosis. These results provided important clues and scientific bases for further population‐based studies investigating whether AFB1 exposure combined with HBV infection would increase the risk of NAFLD. The details of how AFB1 interact with HBx in vivo are still needed to further explore.

The mechanism of the combinative effects of HBx and AFB1 on hepatic lipid metabolism is complex. There have been studies suggesting that the dysregulation of genes involved in de novo lipogenesis, such as sterol regulatory element binding transcription factor 1c (SREBP1C), liver X receptors (LXRs), fatty acid synthase (FASN) and peroxisome proliferator activated receptor gamma (PPARγ), were involved in HBV‐induced hepatic steatosis.^2^ Apart from increasing lipogenesis, HBx up‐regulated fatty acid binding protein 1 (FABP1), a gene responsible intracellular FA transport and utilization.^3^ In addition, mitochondrial dysfunction also has been detected in liver tissues from patients with chronic liver diseases.^37^ The imbalance of fusion and fission homoeostasis is one of the important determinant of mitochondrial function.^38^ In this study, co‐exposure to HBx and AFB1 increased mitochondrial fragmentation in HepG2‐Tet‐ON‐HBx cells, implying changes in mitochondrial dynamics.^25^ Indeed, p‐Drp1^Ser616 ^responsible for the activation of Drp1‐mediated mitochondrial fission were increased in the HBx and AFB1 co‐treated hepatocytes in vitro and ex vivo, as well as the liver of AFB1‐exposed HBV‐Tg mice in vivo. Co‐exposure to HBx and AFB1 also led to a coordinate reduction in the expression of fusion protein Mfn2 in hepatocytes. Mitochondrial dysfunction could impair activity of FA β‐oxidation. Although it was reported that single HBx expression turned out to have no effect on PPARα involved in FA β‐oxidation,^38^ in our study, we found that the protein level of CPT1A, a primary rate‐limiting enzyme involved in mitochondrial FA β‐oxidation and metabolism was down‐regulated in three distinct HBx expressing hepatocytes treated with AFB1. Moreover, the expression of CPT1A was decreased in the AFB1‐exposed PHHs from HBV‐infected HLC mice ex vivo and the liver of HBV‐Tg mice in vivo. Down‐regulation of CPT1A would reduce FA transport into the mitochondrial matrix from the cytoplasm and force FA to LDs for storage in hepatocytes. As a result, co‐exposure to AFB1 and HBx decreased CPT1A, which could exacerbate hepatosis in the hepatocytes. Most importantly, knocking down of Drp1 with si*DNM1L* genetic intervention ameliorated the reduction in CPT1A and the abnormal LDs accumulation in HBx and AFB1 co‐exposed differentiated HepaRG cells, further highlighting that the shift of the mitochondrial dynamics toward fission mediated by Drp1 was involved in HBx and AFB1 co‐exposure‐triggered hepatic steatosis.

For the question of how HBx and AFB1 activated Drp1, our previous investigation into mechanisms of the stemness of nasopharyngeal carcinoma showed that COX‐2 increased the activation of Drp1 by recruiting the mitochondrial translocation of p53.^15^ COX‐2 is a key enzyme in the synthesis of prostaglandins and its expression has been reported to be widely up‐regulated in multiple human cancers, including colorectal cancers,^39^ prostate cancers,^40^ gastric cancers^41^ and pancreatic cancers,^42^ etc, In line with the high expression identified in cancer pathogenesis, this study found that transcription of *PTGS2* gene was up‐regulated in a GEO cohort of HBV‐infected patients. Moreover, both the mRNA and protein level of COX‐2 were increased in the HBx and AFB1 co‐exposed hepatocytes in vitro, the HBV‐infected and AFB1‐exposed PHHs in HLC mice ex vivo, and the liver of AFB1‐treated HBV‐Tg mice in vivo. It was reported that the expression of COX‐2 was extremely low in normal liver,^43^ but was increased under pathological conditions such as acute liver failure,^44^ hepatic fibrosis and cirrhosis,^45^ and hepatocarcinogenesis.^43^ To date, the implication of COX‐2 in hepatic steatosis remains controversial. It was reported that COX‐2 transgenetic mice on a high‐fat diet exhibited reduction in hepatic steatosis and enhancement of insulin sensitivity and glucose tolerance compared with corresponding wild type mice.^46^ But in contrast, expression of COX‐2 was reported to be significantly increased in human hepatic biopsy specimens of patients with NAFLD and the up‐regulation of COX‐2 contributed to protection against insulin resistance.^17^ This finding seemed reasonable since constitutive expression of COX‐2 in hepatocytes could activate proteins involved in cell survival, such as serine/threonine kinase (AKT) and AMP‐activated protein kinase (AMPK), thereby increasing hepatic insulin signalling and preserving insulin resistance during NAFLD.^47^ Conversely, blocking COX‐2 activity with the selective inhibitors, celecoxib or nimesulide, ameliorated steatohepatitis and hyperlipidaemia in transgenic mouse model overexpressing the α1 subunit of AMPK.^48^ Our study supported the facilitating role of COX‐2 in NAFLD by showing that co‐exposure of HBx and AFB1 failed to activate mitochondrial fission protein Drp1 and down‐regulate CPT1A implicated in FA β‐oxidation when COX‐2 was knocked down by si*PTGS2* or CRISPR/Cas9 system (Supporting information: Figure S2), which occurred in parallel to the reduction in LDs.

Drp1‐associated mitochondrial fragmentation is a required step for execution of apoptosis and necroptosis.^49^, ^50^ In this study, combined exposure to AFB1 decreased cell viability in all three HBx‐expressing hepatocytes (Supporting information: Figure S3). Co‐treatment of HBx and AFB1 with Nec‐1 offset the suppression action of HBx and AFB1 on cell viability, implying that such detrimental effects may be related to necroptosis. Indeed, HBx and AFB1 co‐exposure increased the expression of RIP1 and RIP3, as well as the p‐MLKL in hepatocytes. Higher expression of *MLKL* in HBV‐infected patient was correlated with the increased expression of *PTGS2* in these clinical samples. HBx and AFB1‐induced up‐regulation of RIP3 in mitochondrial of HepG2 cells was abrogated when COX‐2 were knocked down by CRISPR/Cas9 system. Therefore, COX‐2 could recruit RIP3 to mitochondria. Most importantly, we verified that RIP3 was in close proximity with COX‐2 on mitochondria in HBx and AFB1‐exposed HepG2‐Tet‐ON‐HBx cells and HepG2 cells (Supporting information: Figure S4). Moreover, co‐precipitated p‐MLKL and RIP3 were significantly reduced after COX‐2 was decreased by si*PTGS2*. These data elucidated that knockdown of COX‐2 protected HBx and AFB1 co‐exposed hepatocytes from necroptosis via suppressing necrosome formation on mitochondria. In addition, we further confirmed that Drp1‐related mitochondrial dynamics abnormalities were also the downstream mediators of necrosome formation during the process of LDs accumulation in HBx and AFB1‐co‐exposed hepatocytes. This is the first study reporting the pivotal role of COX‐2 in regulation of necrosome formation and Drp1‐mediated mitochondrial fragmentation during HBx and AFB1‐induced hepatic steatosis. Therefore, pharmacological modification and genetic intervention targeted at COX‐2 activity seems to be a potential therapeutic strategy counteracting HBV infection and/or AFB1 exposure‐related hepatic steatosis, as well as hepatic diseases associated with necrotic cell death.

**Figure 6:**
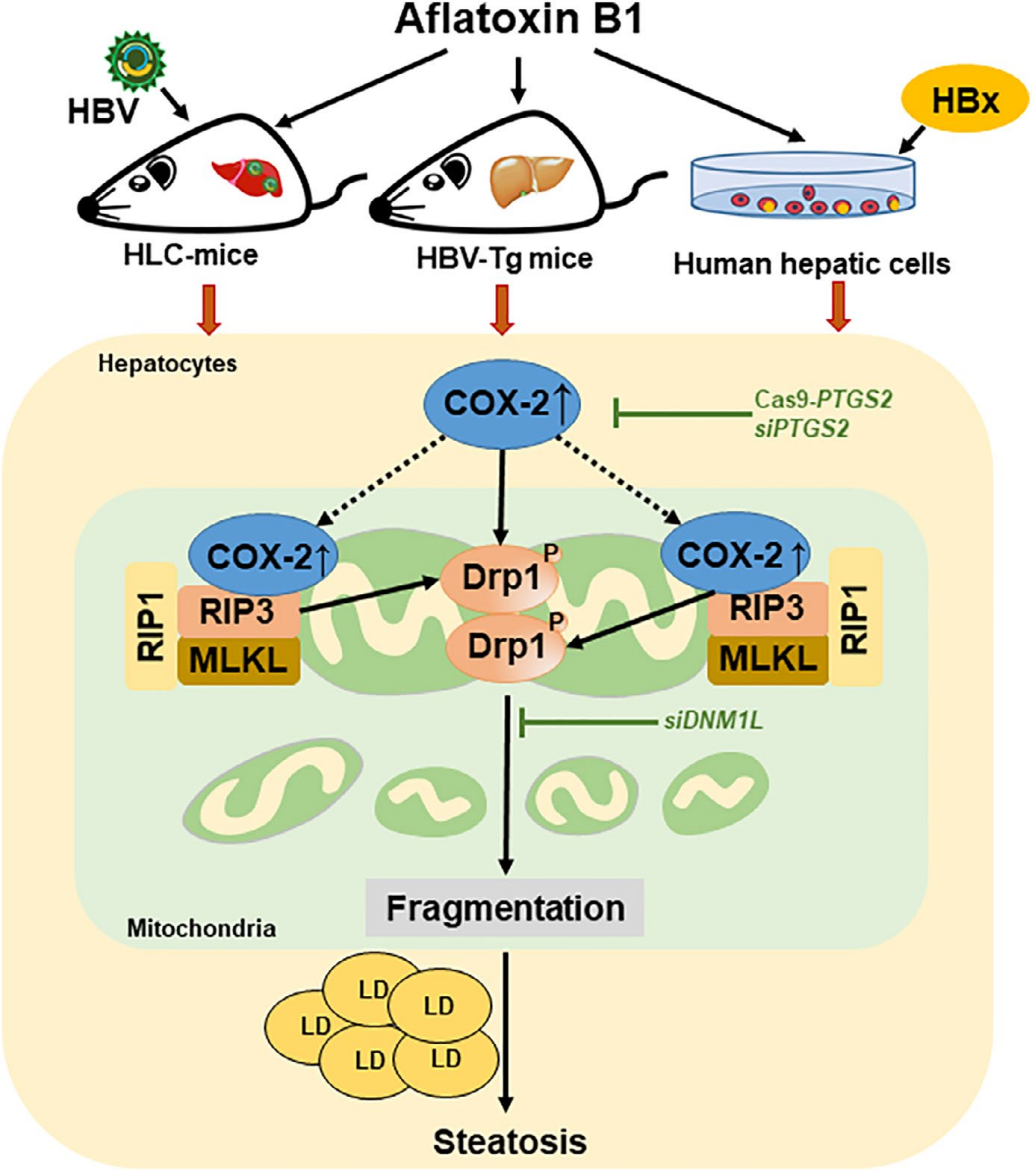
Schematic diagram of the signalling pathways involved in hepatitis B virus X protein (HBx) and aflatoxin B1 (AFB1) co‐exposure‐induced hepatic steatosis. We showed that HBx combined with AFB1 exposure increased amount of lipid droplets (LDs) in the PHHs from HBV‐infected HLC ex vivo, the liver of HBV‐Tg mice in vivo, as well as multiple HBx‐expressing human hepatic cell lines in vitro. In the hepatocytes, AFB1 and HBx increased the expression of COX‐2 in mitochondria and thereby promoting the co‐location of COX‐2 and RIP3‐MLKL necrosome on mitochondria. Moreover, AFB1 and HBx up‐regulated COX‐2 to trigger Drp‐1‐mediated mitochondrial fragmentation and dysfunction in the hepatocytes. Necrosome formation and mitochondrial dynamics disorder finally contributing to the LDs accumulation in the hepatocytes

Taken together, exposure to AFB1, combined with HBx, increased risk of hepatic steatosis. Owing to the up‐regulation of COX‐2, co‐exposure to HBx and AFB1 promoted the formation of RIP3‐MLKL necrosome and induced Drp‐1‐mediated mitochondrial fragmentation and dysfunction in the hepatocytes, finally contributing to the LDs accumulation (Figure 6). COX‐2 could be a target molecular for developing agents for prevention and therapy to the inducible mitochondria‐associated hepatic necroptosis and/or NAFLD.

## CONFLICT OF INTEREST

The authors confirm that there are no conflict of interest.

## AUTHOR CONTRIBUTIONS

LZN and LYC conceived and designed the research; CYY, HPY and JS carried out experiments and analysed the data; C.L and HCY participated in statistical analyses and data interpretation; CYY, LY, HPY, LYC and LZN drafted and revised the manuscript; all authors approved the final manuscript.
