# Supplementary material for: HBx combined with AFB1 triggers hepatic steatosis via COX‐2‐mediated necrosome formation and mitochondrial dynamics disorder
Source: J Cell Mol Med. 2019 Jul 7;23(9):5920–33. doi: 10.1111/jcmm.14388 (PMC6714226; doi:10.1111/jcmm.14388)
Supplement: Supplementary file 1 [file JCMM-23-5920-s001.docx]

**HBx Combined with AFB1 Triggers Hepatic Steatosis via COX-2-mediated Necrosome Formation and Mitochondrial Dynamics Disorder**

Yuan-Yuan Chen^1,*^, Yi Lin^1,^*, Pei-Yu Han^1,2,^*, Shan Jiang^1^, Lin Che^1^, Cheng-Yong He^1^, Yu-Chun Lin^1,^†, Zhong-Ning Lin^1,^†

1. State Key Laboratory of Molecular Vaccinology and Molecular Diagnostics, School of Public Health, Xiamen University, Xiamen, China.

2. Wuxi School of Medicine, Jiangnan University, Wuxi, China

* These authors contributed equally to this work.

**†Corresponding Authors:** Yu-Chun Lin, linych@xmu.edu.cn or Zhong-Ning Lin, linzhn@xmu.edu.cn; State Key Laboratory of Molecular Vaccinology and Molecular Diagnostics, School of Public Health, Xiamen University, Xiang’an South Road, Xiamen, 361102, China.

Tel: +86 592 2880615;

Fax: +86 592 2881578.

**Supporting Information**

1. **Material and methods**

**Generation of HBx-expressing cell lines**

Three HBx-expressing hepatic cell lines including HepG2.2.15 cells integrating two head-to-tail copies of the HBV genome, HBx expressing HepG2 cells (HepG2-Tet-ON-HBx), and differentiated HepaRG cells were selected and constructed. (1) HepG2.2.15 cells were maintained in RPMI-1640 medium (Hyclone, UT, USA) supplemented with 10% fetal bovine serum (FBS) (Gibco, NY, USA). (2) HepG2-Tet-ON-HBx cells were generated using Tet-ON®3G Inducible Expression System (Clontech, CA, USA) as previously described[1], cultured in RPMI-1640 medium containing 10% FBS and were treated with 1 μg/ml of doxycycline (DOX) (Sigma-Aldrich, MO, USA) for 8 h to induce HBx expression. (3) HepaRG cells were cultured in Williams’ E medium (WEM) (Gibco) supplemented with 10% FBS, 4 μg/ml of bovine insulin (Wanbang, Xuzhou, China) and 0.5 mM hydrocortisone hemisuccinate (Sigma-Aldrich) for 2 weeks. For differentiation, it should be seeded at low density for two weeks, and then be cultured in medium supplemented with 1.8% DMSO for another two weeks. For overexpression of HBx, differentiated HepaRG cells were transient transfected with pcDNA3.1-HBX (1 μg/ml) using Lipofectamine® 2000 (Invitrogen, CA, USA) for 12 h[1]. Both HepG2.2.15 and HepaRG cells were obtained from National Institute of Diagnostics and Vaccine Development in Infectious Diseases, Xiamen University.

**Generation of** **COX-2 knockdown cells by CRISPR/cas9 systems**

COX-2 knockdown cell line was generated by CRISPR/Cas9 systems. Oligonucleotide guide RNA (gRNA) sequences directed at the *PTGS2* gene were designed online at http://crispr.mit.edu/ and the sequences were 5′-CACCGCGTTCCAAAATCCCTTGAAG-3′ and 5′-AACCTTCAAGGGATTTTGGAACGC-3′. The gRNA targeting sequences used to generate *PTGS2* knockdown cell line were annealed to synthetic RNA oligonucleotides and subsequently ligated into separate *Esp3I** (Thermo Scientific, MA, USA)-digested lentiCRISPR v2 vector backbones (Addgene 52961, MA, USA). Plasmids were transformed and amplified in *Stbl3* competent cells (Sangon Biotech, Shanghai, China). Then, HEK293T cells were co-transfected with the above-mentioned plasmids and viral packaging plasmids (pCMV-delta 8.9 and VSVG) to produce lentiviral vector stocks. HepG2 cells were infected with lentivirus carrying Cas9-*PTGS2* or Cas9-NC recombinant plasmid using DNA Transfection Reagent X-tremeGENE HP (Roche, Basel, Switzerland). Transfection media was replaced with full media 8 h following transfection for an additional 36 h of resistance marker expression. Finally, Full media was replaced with full media supplemented with 0.8 μg/ml of blasticidin (Gibco) for selection of cells expressing the transfected plasmids.

**Information of antibodies**

Primary antibodies used in immunofluorescence (IF) were: anti-RIP3, Abcam, ab72106, 1;300; anti-COX-2, Abcam, ab52237, 1:200; anti-p-Drp1^Ser616^, Cell Signaling Technology, #3455S, 1:200; anti-TOM20, Cell Signaling Technology, #42406, 1:300; anti-CPT1A, Abcam, Ab128568, 1:300. Secondary antibodies used in IF were: Dylight 405 Goat Anti-Rabbit IgG (H+L) secondary antibody (Beyotime, A0605, 1:500) and Alexa Fluor 488 Goat Anti-Mouse IgG (H+L) secondary antibody (Beyotime, A0423, 1:500).

Primary antibodies used in IHC were: anti-HBx (16F9, National Institute of Diagnostics and Vaccine Development in Infectious Diseases, Xiamen University, 1:500), anti-COX-2 (1:200), anti-RIP3 (1:200) and anti-p-Drp1^Ser616^ (1:200).

Primary antibodies used in Western blot were: anti-p-Drp1^Ser616^, -Drp1, -Mfn1, -Mfn2, -RIP3, and anti-p-MLKL^Ser358^, Abcam, 1:1000; anti-CPT1A, Abcam, 1:2000; anti-RIP1, BD Biosciences, 1:1000; anti-COX-2, Ruiying, 1:1000; anti-GAPDH, Bioworld, 1:10000; anti-COXIV, Beyotime,1:1000. Secondary antibodies used in Western blot were: peroxidase-conjugated goat anti-rabbit IgG (Cell Signaling Technology, #31460) and peroxidase-conjugated anti-mouse IgG (Cell Signaling Technology, #31430).

**Proximity ligation assay (PLA)**

The protein interaction between COX-2 and RIP3 was determined by PLA using a Duolink® In Situ Detection Reagents (Sigma-Aldrich). The cells were fixed on the slides after treatment. The slides were blocked in Duolink II solution for 1 h and were incubated with anti-COX-2 (1:300) or anti-RIP3 (1:300) primary antibody at 4℃ overnight. Then, the slides were incubated with Duolink PLA anti-Mouse PLUS and PLA anti-Rabbit PLUS proximity probes for 1 h at 37°C. After washing, the slides were incubated with ligation-ligase solution for 30 min at 37°C followed by incubation with amplification-polymerase solution for an additional 100 min at 37°C. Finally, the slides were mounted using Mounting Medium with DAPI (Sigma-Aldrich) and the images were captured using confocal microscope. The quantification of PLA signals was performed using IPP 6.0 software.

**Cell viability assay**

Cell viability was measured using the MTS assay (Promega, MW, USA) according to the manufacturer^，^s instructions. At the end of treatment, the cells were incubated with 0.5 mg/ml MTS for 4 h at 37°C. The resulting formazan crystals were dissolved with DMSO and the absorbance was read at 490 nm. The OD values of untreated control cells were set at 100% and the values for treated cells were expressed as % of untreated controls.

1. **Supplementary Tables**

**Table S1 Primers used for qRT-PCR in this study.**

| **Gene name** | **Amplicon (bp)** | **5’-primer** | **3’-primer** | **Accession number** |
| --- | --- | --- | --- | --- |
| *PTGS2* | 285 | CAGCCATACAGCAAATCCTTG | CAAATGTGATCTGGATGTCAAC | NM_000963.3 |
| *CYP1A1* | 198 | GCTCCTGGCTGTCACCGTAT | GCCCTTGAAGTCATCTCCCT | NM_000499.5 |
| *CYP2B6* | 130 | GAGTGTGGAGGA GCGGAT T | AGACGATGGAGCAGATGATGT | NM_000767.5 |
| *CYP3A4* | 186 | GTTCCTCCCTGA AAGATTC | GGGGATCTGTGTTTCTTTAC | NM_001202855.2 |
| *HNF4A* | 172 | ACATGTACTCCTGCAGAT | GAGGTGATCTGTCGGGA | NM_000457 |
| *ALB* | 225 | TACAAATTCCAGAATGCGCT | TTCAGGACCACGGATAGAT | [NM_000477.6](https://www.ncbi.nlm.nih.gov/nuccore/NM_000477.6) |
| *ACTB* | 256 | CACCAGGGCGTGATGGT | CTCAAACATGATCTGGGTCAT | NM_001101.4 |

*PTGS2*, prostaglandin-endoperoxide synthase 2; *CYP1A1*, cytochrome P450 family 1 subfamily A member 1; *CYP2B6*, cytochrome P450 family 2 subfamily B member 6; *CYP3A4*, cytochrome P450 family 3 subfamily A member 4; *HNF4A*, hepatocyte nuclear factor 4*α*; *ALB*, albumin; *ACTB*, actin beta.

1. **Supplementary Figures**

**Figure S1**

**
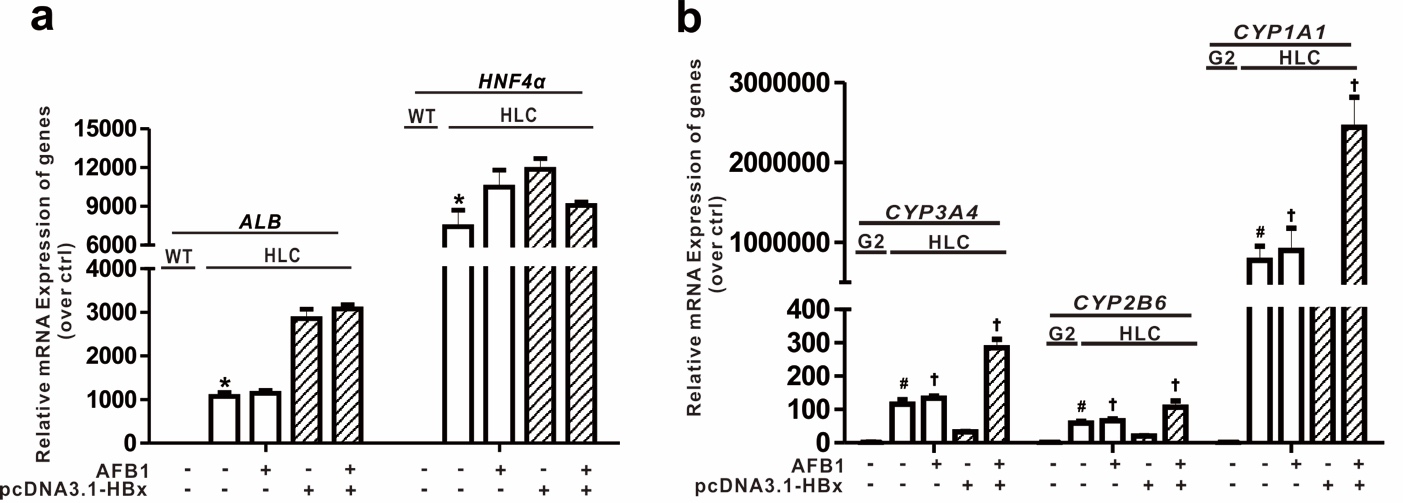
**

**Fig. S1 Generation of the HLC mice. (a)** The mRNA expression of *ALB* and *HNF4A* was increased in PHHs from HLC mice compared with those in primary hepatocytes from WT mice. **(b)** The mRNA level of xenobiotic metabolizing enzymes. The mRNA expression of *CYP3A4*, *CYP2B6*, and *CYP1A1* were increased in PHHs from HLC mice compared with those in the HepG2 cells. G2: HepG2 cells. Our data showed upregulation of CYPs were induced by AFB1 treatment, indicating hepatotoxicity may due to the activation of AFB1 metabolism in hepatic cells. Data were mean ± SD. n=3. * *P* < 0.05 compared with WT mice. ^#^ *P* < 0.05 compared with HepG2 cells. † *P* < 0.05 compared with unexposed PHHs.

**Figure S2**


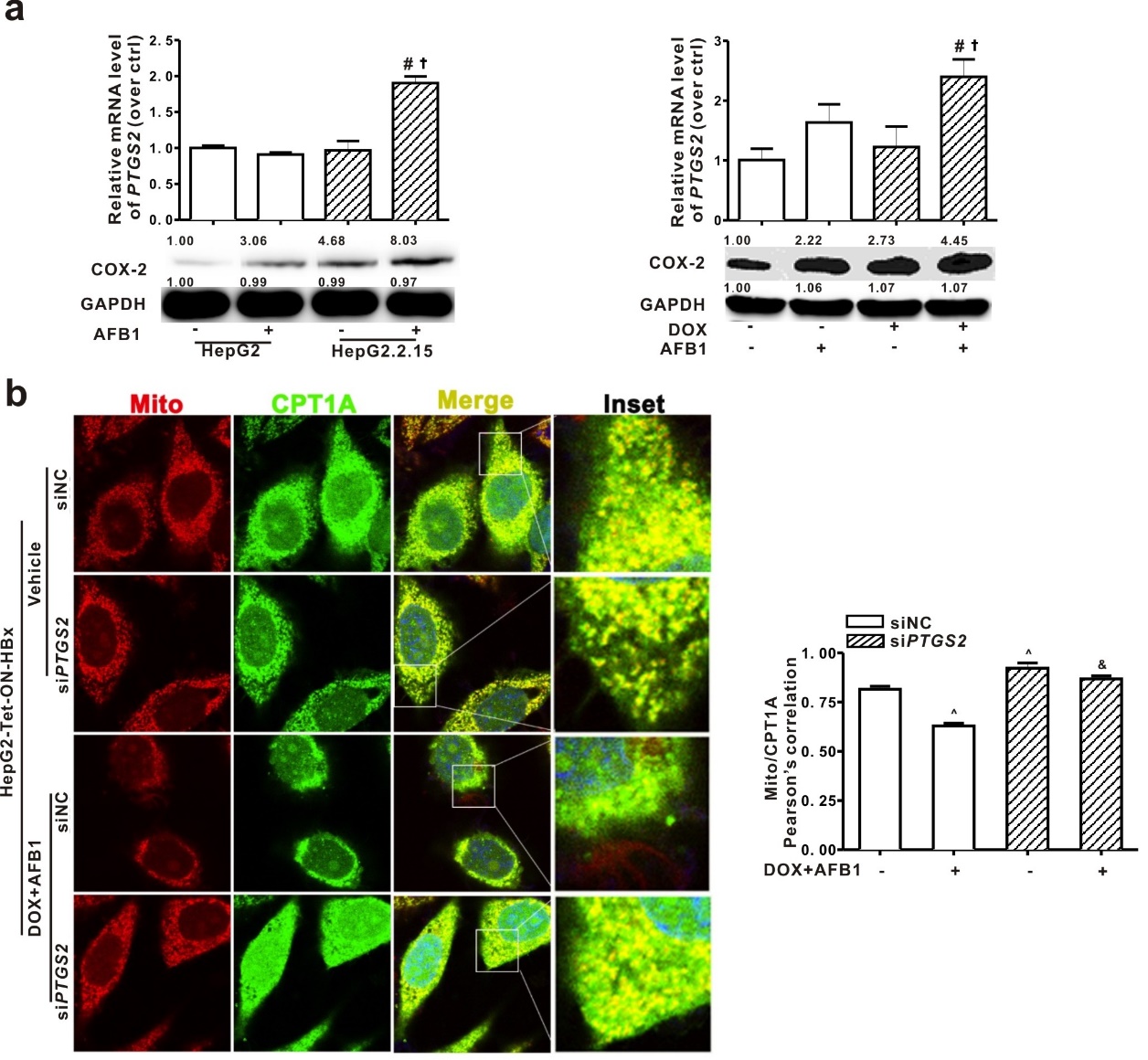


**Fig. S2** **HBx and AFB1 co-treatment decreased CPT1A via COX-2.** **(a)** The mRNA (Upper) and protein (Lower) levels of COX-2 in HepG2.2.15 and HepG2-Tet-ON-HBx cells (Left). The mRNA (Upper) and protein (Lower) expression of COX-2 was increased in both the AFB1-exposed HepG2.2.15 cells and the AFB1-exposed HepG2-Tet-ON-HBx cells upon DOX induction compared with corresponding control cells (Right). For western blot, GAPDH was served as a loading control; and for mRNA expression, *ACTB* was served as a loading control. **(b)** HepG2-Tet-ON-HBx cells were transfected with siNC or si*PTGS2* (50 nM) for 12 h before DOX stimulation and AFB1 treatment. Representative images (Left) and spectrophotometric quantification (Right) of the immunofluorescence co-staining for MitoTracker (Red) and CPT1A (Green) in HepG2-Tet-ON-HBx cells measured by confocal microscopy. Scale bar represents 10 μm. HBx and AFB1 co-treatment resulted in a decreased protein expression of CPT1A in the mitochondria of HepG2-Tet-ON-HBx cells. HBx and AFB1-stimulated downregulation of CPT1A were ameliorated when COX-2 was knocked down by si*PTGS2* in the HepG2-Tet-ON-HBx cells*.* Data were mean ± SD. ^#^ *P* < 0.05 compared with HepG2.2.15 cells or DOX-induced HepG2-Tet-ON-HBx cells. † *P* < 0.05 compared with AFB1-exposed HepG2 cells or AFB1-exposed HepG2-Tet-ON-HBx cells. ^ *P* < 0.05 compared with HepG2-Tet-ON-HBx cells transfected with siNC. ^&^ *P* < 0.05 compared with HBx and AFB1 co-treated HepG2-Tet-ON-HBx cells transfected with siNC.

**Figure S3**


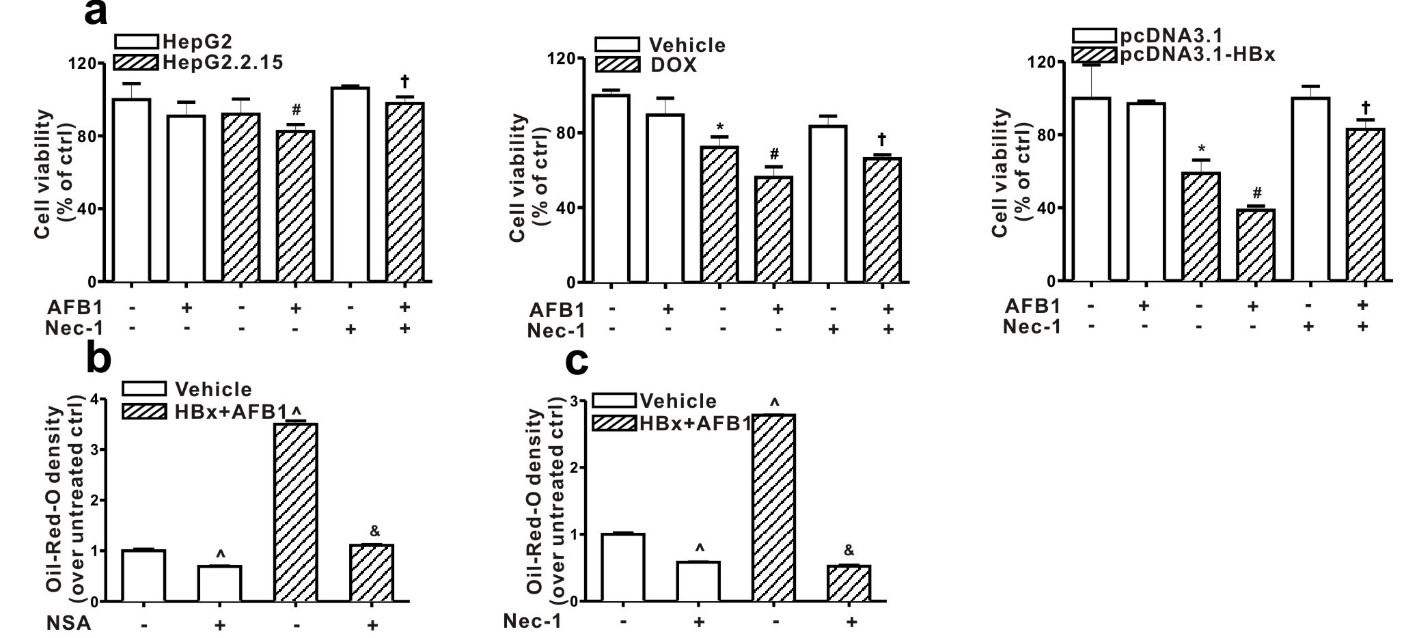


**Fig. S3. HBx combined with AFB1 exposure decreased cell viability in human hepatic cells.** **(a)** Three human hepatic cell lines, including HepG2.2.15 (Left), HepG2-Tet-ON-HBx (Middle), and differentiated HepaRG cells (Right), were used. The viability of three HBx-expressing cells with AFB1 and/or Nec-1 treatment. Combined exposure to AFB1 decreased cell viability in all HBx-expressing hepatocytes. Co-treatment of HBx and AFB1 with necroptosis inhibitor Nec-1 offset the suppression action of HBx and AFB1 on cell viability. **(b)** Spectrophotometric quantification of Oil-Red-O density for lipid droplets shown in Fig. 4h. **(c)** Spectrophotometric quantification of Oil-Red-O density for lipid droplets shown in Fig. 4i. Pretreatment of NSA (b and Fig. 4h) or Nec-1 (c and Fig. 4i) counteracted the increase of lipid droplets in the differentiated HepaRG cells by HBx and AFB1 exposure. Data were mean ± SD. n=3. * *P* < 0.05 compared with corresponding unexposed cells. ^#^ *P* < 0.05 compared with corresponding HBx-expressing cells. † *P* < 0.05 compared with corresponding cells treated with Nec-1. ^ *P* < 0.05 compared with differentiated HepaRG cells treated with NSA or Nec-1 vehicle. ^&^ *P* < 0.05 compared with HBx and AFB1 co-treated differentiated HepaRG cells.

**Figure S4**


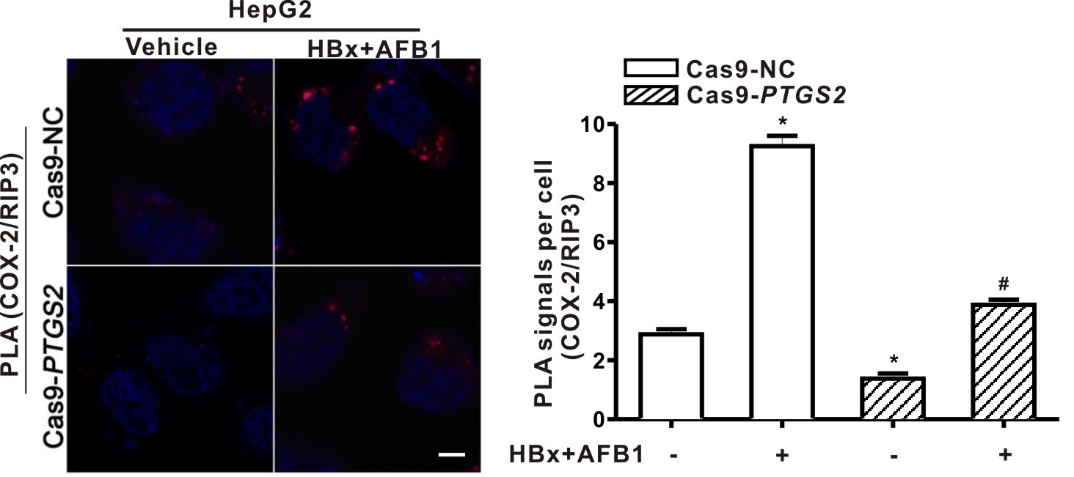


**Fig. S4.** **The interaction of COX-2 with RIP3 detected by PLA in HepG2-Cas9-NC and HepG2-Cas9-*PTGS2* cells.** Interaction events were shown as red foci (Left). Scale bar represents 10 μm. Quantification of COX-2 and RIP3 interaction events were shown on the bar graph (Right). HBx and AFB1 co-treatment increased the interaction of COX-2/RIP3 in HepG2-Cas9-NC and HepG2-Cas9-*PTGS2* cells, whereas the interaction was reduced when COX-2 was knocked down by CRISPR/Cas9 system in HepG2-Cas9-*PTGS2* cells. Data were mean ± SD. n=3. ** P*< 0.05 compared with HepG2-Cas9-NC cells. ^#^ *P* < 0.05 compared with HBx and AFB1-cotreated HepG2-Cas9-NC cells.

**Reference**

1. **He C, Qiu Y, Han P, Chen Y, Zhang L, Yuan Q, Zhang T, Cheng T, Yuan L, Huang C, Zhang S, Yin Z, Peng XE, Liang D, Lin X, Lin Y, Lin Z, Xia N.** ER stress regulating protein phosphatase 2A-B56gamma, targeted by hepatitis B virus X protein, induces cell cycle arrest and apoptosis of hepatocytes. *Cell death & disease*. 2018; 9: 762.
